# Racial disparities in triple negative breast cancer: toward a causal architecture approach

**DOI:** 10.1186/s13058-022-01533-z

**Published:** 2022-06-01

**Authors:** Scott D. Siegel, Madeline M. Brooks, Shannon M. Lynch, Jennifer Sims-Mourtada, Zachary T. Schug, Frank C. Curriero

**Affiliations:** 1 Helen F. Graham Cancer Center and Research Institute, Christiana Care Health System, 4701 Ogletown-Stanton Road, Newark, DE 19713 USA; 2 Institute for Research on Equity and Community Health, Christiana Care Health System, Newark, USA; 3 Fox Chase Cancer Center, Philadelphia, USA; 4 The Wistar Institute Cancer Center, Philadelphia, USA; 5 Department of Epidemiology, Johns Hopkins Spatial Science for Public Health Center, Johns Hopkins Bloomberg School of Public Health, Baltimore, USA

**Keywords:** Triple negative breast cancer, Racial disparities, Neighborhood effects, Alcohol, Obesity

## Abstract

**Background:**

Triple negative breast cancer (TNBC) is an aggressive subtype of invasive breast cancer that disproportionately affects Black women and contributes to racial disparities in breast cancer mortality. Prior research has suggested that neighborhood effects may contribute to this disparity beyond individual risk factors.

**Methods:**

The sample included a cohort of 3316 breast cancer cases diagnosed between 2012 and 2020 in New Castle County, Delaware, a geographic region of the US with elevated rates of TNBC. Multilevel methods and geospatial mapping evaluated whether the race, income, and race/income versions of the neighborhood Index of Concentration at the Extremes (ICE) metric could efficiently identify census tracts (CT) with higher odds of TNBC relative to other forms of invasive breast cancer. Odds ratios (OR) and 95% confidence intervals (CI) were reported; *p*-values < 0.05 were significant. Additional analyses examined area-level differences in exposure to metabolic risk factors, including unhealthy alcohol use and obesity.

**Results:**

The ICE-Race, -Income-, and Race/Income metrics were each associated with greater census tract odds of TNBC on a bivariate basis. However, only ICE-Race was significantly associated with higher odds of TNBC after adjustment for patient-level age and race (most disadvantaged CT: OR = 2.09; 95% CI 1.40–3.13), providing support for neighborhood effects. Higher counts of alcohol and fast-food retailers, and correspondingly higher rates of unhealthy alcohol use and obesity, were observed in CTs that were classified into the most disadvantaged ICE-Race quintile and had the highest odds of TNBC.

**Conclusion:**

The use of ICE can facilitate the monitoring of cancer inequities and advance the study of racial disparities in breast cancer.

**Supplementary Information:**

The online version contains supplementary material available at 10.1186/s13058-022-01533-z.

## Introduction

Breast cancer mortality rates are 40% higher for Black than White women in the US (28.2 vs. 20.1 per 100,000) despite similar incidence rates (127.3 vs. 131.6 per 100,000) [1]. Multiple risk factors are thought to drive this Black–White disparity [2, 3], including racial differences in insurance status, tumor characteristics, comorbidities, and treatment quality [4, 5]. However, traditional risk factor approaches typically do not consider the larger context within which these risk factors operate (e.g., neighborhood effects). For example, insurance status predicts metabolic outcomes (e.g., obesity) [6, 7] and, in turn, metabolic outcomes have been found to place Black women at a greater risk for more aggressive subtypes of breast cancer [8] and higher breast cancer mortality rates [9]—all of which can be exacerbated by residing in neighborhoods with limited healthy retail food options [10]. These findings call into question the validity of investigating risk factors separate from neighborhood circumstances.

The neighborhood context is particularly relevant in the context of triple negative breast cancer (TNBC). TNBC is an aggressive subtype of invasive breast cancer with twice the incidence rates for Black relative to White women [11, 12]. Compared to other invasive breast cancer subtypes, TNBC is more likely to present at a younger age (often before screening mammography is recommended), between screening mammograms (i.e., interval cancers), and at a more advanced stage [13], underscoring the critical need for improved prevention and early detection. As reviewed elsewhere, several potential patient-level risk factors for TNBC have been identified, with varying levels of supporting evidence, including reproductive (age at menarche and menopause, parity, breastfeeding), metabolic (obesity, type 2 diabetes, alcohol use), and genetic (*BRCA1*, *BRCA2*) factors [11, 13, 14]. More recent studies have found that area-level measures of socioeconomic status (SES) are inversely associated with TNBC risk, even after adjusting for patient characteristics, providing support for neighborhood effects [15–17]. Further, neighborhood effects have been found to aid in improving the targeting of prevention and early detection interventions by identifying areas with a high cancer burden that could be attributable to potentially modifiable risk factors. [18]

Viewed through a *causal architecture* framework, neighborhood effects can be conceptualized as a system of exposure that contributes to variations in TNBC risk across residentially segregated populations [19, 20]. In contrast to a traditional risk factor approach, a causal architecture approach would aim to clarify the network of causes that contribute to breast cancer disparities [20]. That is, rather than attempting to estimate the population-wide effects of individual risk factors, a greater emphasis would be placed on understanding how multiple, co-occurring exposures work together to produce different rates of disease between populations. Furthermore, more attention would be paid to underlying structures that explain systems of exposure [19]. For example, structural racism contributes to residential segregation and areas of disinvestment, yielding quite different systems of exposure between neighborhoods (e.g., access to employer-based insurance, high-quality health care, healthy food, etc.). [21]

New approaches are needed to efficiently identify systems of exposure that account for racial disparities in TNBC. Toward that end, the primary objective of this study was to test whether the Index of Concentration at the Extremes (ICE) metric could identify neighborhood-level systems of exposure associated with risk for TNBC in New Castle County, Delaware. Analyses were focused on this geographic region because Delaware has among the highest TNBC incidence rates in the US [22], with more cases concentrated in New Castle County relative to the other two counties in the state [23]. The ICE metric quantifies the degree to which residents within a geographic unit (e.g., census tracts) are concentrated into segregated groups of extreme disadvantage and advantage [24, 25]. Three versions of ICE can be calculated based on income, race, and both race and income. Krieger and colleagues have observed that the ICE-Race/Income metric generally outperforms the other ICE metrics when predicting health disparities [25, 26]. The ICE-Race/Income metric differs from other commonly employed indices, such as the Yost index or Area Deprivation Index (ADI) [27, 28], in at least two respects. First, the ICE metric represents a measure of social inequality by incorporating information on both disadvantage and advantage, rather than disadvantage alone. Second, the ICE-Race/Income metric operationalizes social inequality with both race and SES data, rather than SES data alone. The ICE-Race/Income metric offers the added benefit of being robust to multicollinearity, a statistical challenge frequently encountered in studies that included measures of segregation for both income and race [29]. Prior research has observed a link between ICE-Income and overall breast cancer survival [30] and ICE-Income, -Race, and -Income/Race and the odds of estrogen receptor status [29], but has not been investigated in the context of TNBC. Given the higher TNBC incidence rate observed for Black women and the relationship between TNBC and spatial measures of SES, we hypothesized that all three ICE metrics would be associated with the spatial odds of TNBC, with the greatest odds observed for the ICE-Race/Income metric.

The secondary objective of this study was to test for cross-level interactions between patient-level race and the ICE-Race metric. Prior findings that have suggested Black women living in low-SES but predominantly White neighborhoods experienced a greater risk of TNBC relative to Black women in low-SES predominantly Black neighborhoods [15]. We hypothesized that higher rates of social inequality, as measured by the ICE metrics, would be associated with greater odds of TNBC.

The tertiary objective of this study was to conduct a sensitivity check on the utility of the ICE metrics to efficiently identify neighborhoods with systems of exposure relevant to breast cancer risk. Specifically, we evaluated whether the ICE metrics were associated with metabolic risk factors, including census tract measures of alcohol and fast-food retailers, unhealthy alcohol use, and obesity. While alcohol is an established risk factor for certain breast cancer subtypes [31], its link with TNBC specifically is less clear [32]. When investigated in cohorts stratified by race, however, alcohol use has been shown to be positively associated with TNBC risk in Black but not White women [33, 34]. This would suggest that alcohol is not necessarily a ubiquitous risk factor for TNBC but that the presence of additional factors that covary with race, such as neighborhood characteristics, moderate the relationship between alcohol use and TNBC risk. Compared to White women, Black women are more likely to be exposed to racial discrimination [35], interpersonal abuse [36], and neighborhoods with elevated alcohol retailer density [37–39], which have all been associated with binge drinking and other patterns of unhealthy alcohol use [40]. Binge drinking predicts increased breast cancer risk even after adjusting for lifetime alcohol intake [41]. Unhealthy alcohol use may also interact with other neighborhood exposures that disproportionately affect Black women, such as limited healthy food options and its connection to obesity and metabolic syndrome [42, 43]. Metabolic syndrome has been shown to mediate nearly half the racial disparity in TNBC incidence [44]. Therefore, we hypothesized that the ICE metrics would be associated with greater exposure to metabolic risk factors.

## Methods

### Setting

Patient records came from the Helen F. Graham Cancer Center and Research Institute (HFGCCRI) cancer registry, a part of the Christiana Care Health System and based in New Castle County, Delaware. The HFGCCRI provides care to an average of more than 600 breast cancer cases annually. As detailed elsewhere, the HFGCCRI breast cancer population accounts for 85% of all cases from the surrounding county and are representative of the county population of cases in terms of age, race, receptor status, and stage [45].

### Study population

This study population consisted of 3316 adult female New Castle County residents who were diagnosed with invasive breast cancer between the years of 2012 and 2020. To better understand Black–White disparities, the population was limited to women who self-reported as either Black (*n* = 776) or White (*n* = 2540), regardless of ethnicity. The time frame was selected to maximize the number of breast cancer cases where the subtype markers necessary for classifying patients with TNBC were routinely documented in the cancer registry. Patient residential address, demographic, insurance payer, and clinical data were abstracted from the registry. Patient addresses were manually cleaned and geocoded using ArcGIS 10.8 [46], yielding a match rate of 95% (3316/3484). Of the 168 unmatched records, 114 geocoded to another county, two geocoded to out of state, 47 had PO box addresses, three had missing address information, and two could not be located. Unmatched patients did not significantly differ from matched patients by age, race, ethnicity, stage, subtype, or insurance payer.

### Patient measures

Demographic measures included age at diagnosis, race, and insurance payer status, which were all directly abstracted from the HFGCCRI cancer registry. Insurance payer status (private/commercial, Medicaid, Medicare, none, or unknown) was used as a proxy for access to health care and socioeconomic status [47]. Clinical measures included breast cancer stage and receptor status. Cases were classified into ‘TNBC’ when the receptors for estrogen (ER), progesterone (PR), and human epidermal growth factor 2 (HER2) were all known negative; all other invasive cases were classified as ‘Non-TNBC.’

### Census tract measures

New Castle County is subdivided into 130 census tracts, which provide stable geographic units for reporting population statistics [48]. All census tract sociodemographic data were obtained from the US Census Bureau’s American Community Survey 2014–2018 5-year estimates [49]. ICE-Income, -Race, and -Income/Race metrics were calculated for all New Castle County census tracts according to the following general formula [24, 25]:

1 $${\text{ICE}}_{i} = (A_{i} {-}D_{i} ){/}T_{i}$$

where *A*_*i*_ is the number of advantaged persons in a census tract, *D*_*i*_ is the number of disadvantaged persons in a census tract, and *T*_*i*_ is the total population in the census tract *i*. For ICE-Income, advantaged and disadvantaged were defined as households with income ≥ $125,000 or < $20,000. For ICE-Race, advantaged and disadvantaged were defined as non-Hispanic White and non-Hispanic Black. For ICE-Race/Income, advantaged and disadvantaged were defined as non-Hispanic White households with income ≥ $125,000 and non-Hispanic Black households with income < $20,000. ICE values for geographic units range from − 1, indicating that 100% of the population can be classified into the most disadvantaged group, to + 1, indicating that 100% of the population can be classified into the most advantaged group. All ICE measures were classified into quintiles based on their distribution within New Castle County, setting Q5 (most advantaged) as the reference group.

Area-level measures were used to estimate the potential impact of environmental or neighborhood factors on rates of obesity and unhealthy alcohol use, similar to the conceptualization of ‘obesogenic’ environments [50]. Census tract prevalence measures of obesity and disordered alcohol use were generated from Christiana Care Health System electronic health record (EHR) data for 20,310 unique adult New Castle County residents who were admitted to an inpatient unit between July 1, 2018 and June 30, 2019, regardless of admitting diagnosis or demographics. Previous work has shown that such measures generated from inpatient data are generally representative of risk factor prevalence among New Castle County census tracts [51]. International Classification of Diseases (ICD) diagnosis codes abstracted from the EHR for obesity and alcohol use disorder (AUD) were used to categorize patients into ‘obese’ or ‘not obese’ and ‘AUD’ or ‘no AUD’ categories. Consistent with clinical guidelines [52], obesity was defined as a BMI of ≥ 30. AUD diagnoses were made by treating physicians and based on the *Diagnostic and Statistical Manual of Mental Disorders* (5th edition) [53] criteria, which assess clinically significant, unhealthy patterns of use (e.g., large quantities, cravings, tolerance, withdrawal). Patient addresses were manually cleaned and geocoded using ArcGIS 10.6, yielding a match rate of 98% (20,310/20,706). Patient-level data on obesity and alcohol use were not available for the breast cancer study population.

Census tract measures of fast-food restaurants and alcohol retailers in New Castle County were produced from commercial data and publicly available records. Fast-food retailer data were obtained from SICCODE.com, utilizing the North American Industry Classification System (NAICS) code 722513, [54] consistent with established approaches [55]. Alcohol retailer data were drawn from a public state business license database that was current as of April 17, 2019 [56]. Guided by studies that have more reliably observed a relationship between disordered alcohol use and residential exposure to off-premise alcohol retailers (e.g., liquor stores), but not on-premise alcohol retailers (e.g., bars) [57], we included only off-premise retailers. All retail locations were geocoded using ArcGIS 10.8 [46] with a match rate of 100% (fast-food retailer *N* = 221, alcohol retailer *N* = 160).

### Statistical analyses

Spatial data management and statistical analyses were performed in the R Statistical Computing Environment using various packages [58–63]. Descriptive and bivariate statistics, and post hoc tests with Bonferroni-adjusted *p*-values, were used to compare TNBC versus Non-TNBC patient groups by the sociodemographic, clinical, and ICE variables derived from patient and census tract measures.

Multilevel logistic regression models were used to examine the odds of TNBC (vs. Non-TNBC) before and after adjusting for patient (level-1) and census tract (level-2) variables. The multilevel logistic regression model included a census tract-level random effect to account for the clustering of patients within tracts. Patient-level variables included age at diagnosis, race (Black, White), and insurance (commercial, Medicaid/none). Tract-level variables included the ICE-Race, -Income, and -Race/Income quintiles.

Three univariate and multivariate models tested each ICE measure separately, with all models adjusting for patient-level age at diagnosis and race. Additional multivariate models tested cross-level interactions between patient-level race and tract-level ICE quintiles. Based on the results of these models, details of which are provided in the results, multivariate logistic regression models were stratified by Black and White race to examine differential effects of tract-level ICE-Race on odds of TNBC after adjustment for age of diagnosis. Odds ratios and 95% confidence intervals were reported; *p*-values less than 0.05 were significant.

The spatial covariation of TNBC and ICE measures were visualized using bivariate choropleth maps. First, breast cancer patients were aggregated to their census tract of residence to create tract-level measures of the percentage of patients with TNBC. The % TNBC and ICE values were separated into quintiles based on their respective tract-level distributions within New Castle County. For the ICE measures, quintiles were coded such that lower ICE values (representing greater disadvantage) correspond to higher quintiles representing greater relative disadvantage. The quintiles of % TNBC and ICE were combined to create 5 × 5 classification systems that denote whether census tracts are relatively low, moderate, or high in each value. The resulting 25 classification values were symbolized using color and saturation to simultaneously show variation in both measures. For ease of visualization, only the highest/lowest quintile extremes of the classification system (low/low, low/high, high/low, and high/high) were colored in the maps. Geocoding and final map preparations were conducted in ArcMap 10.8 [46].

To begin to characterize place-based systems of exposure related to metabolic risk factors for TNBC, descriptive tables were created where census tracts were classified according to their quintiles of TNBC and ICE-Race (low/low, low/high, high/low, and high/high) that were visualized in the bivariate choropleth map. Population data from the American Community Survey were used to describe race, poverty, and education levels for the census tract groups [49]. Tract-level data on systems of exposure included alcohol and fast-food retailers, as well as prevalence of AUD and obesity. Supplemental bar charts show the variation of alcohol retailers, fast-food retailers, AUD prevalence, and obesity prevalence by census tract ICE quintiles.

## Results

TNBC cases accounted for 14% of the invasive breast cancer cases in the study population (Table 1). Compared to those without TNBC, TNBC cases were significantly younger at diagnosis (mean age 60.2 vs. 63.0), twice as likely to be Black (39.5% vs. 20.9%), more likely to have Medicaid or no insurance (8.2% vs. 5.4%), less likely to have Medicare (35.8% vs. 42.2%), and twice as likely to present with a late-stage cancer (14.8% vs. 7.2%). Comparing ICE measures by census tract of residence, TNBC cases were significantly overrepresented among Q1 (the most disadvantaged quintile) tracts for ICE-Race (23.0% vs. 12.8%), ICE-Income (17.7% vs. 12.5%), and ICE-Race/Income (18.1% vs. 11.5%). TNBC cases were similarly underrepresented among Q5 (the most advantaged quintile) tracts across all ICE measures.

**Table 1:** Characteristics of Black and White breast cancer patients by cancer subtype, New Castle County, DE

	TNBC (*N* = 453)	Non-TNBC (*N* = 2863)	Total (*N* = 3316)	*p*-values
Age at diagnosis, mean (SD)**	60.2 (14.6)	63.0 (13.2)	62.6 (13.4)	< 0.001
Race, *n* (%)**				< 0.001
White	274 (60.5)	2266 (79.1)	2540 (76.6)	–
Black/African American	179 (39.5)	597 (20.9)	776 (23.4)	–
Insurance, *n* (%)*				0.015
Private	251 (55.4)	1470 (51.3)	1721 (51.9)	0.468
Medicare^†^	162 (35.8)	1207 (42.2)	1369 (41.3)	0.041
Medicaid/none	37 (8.2)	156 (5.4)	193 (5.8)	0.120
Unknown	3 (0.7)	30 (1.0)	33 (1.0)	1.000
Stage of diagnosis^a^, *n* (%)**				< 0.001
Early stage^††^	381 (84.1)	2637 (92.1)	3018 (91.0)	< 0.001
Late stage^††^	67 (14.8)	205 (7.2)	272 (8.2)	< 0.001
Unknown stage	5 (1.1)	21 (0.7)	26 (0.8)	1.000
Census tract ICE-Race, *n* (%)**				< 0.001
Q1^††^ (most disadvantaged)	104 (23.0)	367 (12.8)	471 (14.2)	< 0.001
Q2	102 (22.5)	585 (20.4)	687 (20.7)	1.000
Q3	103 (22.7)	694 (24.2)	797 (24.0)	1.000
Q4	95 (21.0)	621 (21.7)	716 (21.6)	1.000
Q5^††^ (most advantaged)	49 (10.8)	596 (20.8)	645 (19.5)	< 0.001
Census tract ICE-Income, *n* (%)**				< 0.001
Q1^††^ (most disadvantaged)	80 (17.7)	359 (12.5)	439 (13.2)	0.028
Q2	96 (21.2)	487 (17.0)	583 (17.6)	0.298
Q3	64 (14.1)	475 (16.6)	539 (16.3)	1.000
Q4	114 (25.2)	699 (24.4)	813 (24.5)	1.000
Q5^††^ (most advantaged)	99 (21.9)	843 (29.4)	942 (28.4)	0.009
Census tract ICE-Race/Income, *n* (%)**				< 0.001
Q1^††^ (most disadvantaged)	82 (18.1)	329 (11.5)	411 (12.4)	0.001
Q2	112 (24.7)	547 (19.1)	659 (19.9)	0.054
Q3	77 (17.0)	562 (19.6)	639 (19.3)	1.000
Q4	94 (20.8)	693 (24.2)	787 (23.7)	1.000
Q5^††^ (most advantaged)	88 (19.4)	732 (25.6)	820 (24.7)	0.049

^a^Early stage includes stages 1-3a and late stage includes stages 3b-4

^*^Significant at *p* < 0.05

^**^Significant at *p* < 0.001

^†^Post-hoc test significant at *p* < 0.05

^††^Significant at *p* < 0.001

See Table 2 for descriptive statistics on TNBC cases stratified by race. Compared to White TNBC cases, Black TNBC cases were significantly younger (mean age 56.9 vs. 62.4) and more likely to have private insurance (63.7% vs. 50.0%) but less likely to have Medicare (24.0% vs. 43.4%). Differences in insurance status can likely be attributed to mean age differences between Black and White TNBC cases. No significant differences were observed for stage of diagnosis. Comparing ICE measures by census tract of residence, Black TNBC cases were significantly overrepresented among Q1 (the most disadvantaged quintile) tracts for ICE-Race (43.6% vs. 9.5%), ICE-Income (31.3% vs. 8.8%), and ICE-Race/Income (36.3% vs. 6.2%). Black TNBC cases were similarly underrepresented among Q5 (the most advantaged quintile) tracts across all ICE measures.

**Table 2:** Characteristics of patients with triple negative breast cancer by race, New Castle County, DE

	Black (*N* = 179)	White (*N* = 274)	Total (*N* = 453)	*p*-values
Age at diagnosis, mean (SD)**	56.9 (13.1)	62.4 (15.2)	60.2 (14.6)	< 0.001
Insurance, *n* (%)**				< 0.001
Private^†^	114 (63.7)	137 (50.0)	251 (55.4)	0.020
Medicare^††^	43 (24.0)	119 (43.4)	162 (35.8)	< 0.001
Medicaid/None	20 (11.2)	17 (6.2)	37 (8.2)	0.313
Unknown	2 (1.1)	1 (0.4)	3 (0.7)	1.000
Stage of diagnosis^a^, *n* (%)				0.857
Early stage	153 (85.5)	228 (83.2)	381 (84.1)	–
Late stage	24 (13.4)	43 (15.7)	67 (14.8)	–
Unknown stage	2 (1.1)	3 (1.1)	5 (1.1)	–
Census tract ICE-Race, *n* (%)**				< 0.001
Q1^††^ (most disadvantaged)	78 (43.6)	26 (9.5)	104 (23.0)	< 0.001
Q2	46 (25.7)	56 (20.4)	102 (22.5)	1.000
Q3	37 (20.7)	66 (24.1)	103 (22.7)	1.000
Q4^††^	15 (8.4)	80 (29.2)	95 (21.0)	< 0.001
Q5^††^ (most advantaged)	3 (1.7)	46 (16.8)	49 (10.8)	< 0.001
Census tract ICE-Income, *n* (%)**				< 0.001
Q1^††^ (most disadvantaged)	56 (31.3)	24 (8.8)	80 (17.7)	< 0.001
Q2	43 (24.0)	53 (19.3)	96 (21.2)	1.000
Q3	23 (12.8)	41 (15.0)	64 (14.1)	1.000
Q4	38 (21.2)	76 (27.7)	114 (25.2)	1.000
Q5^††^ (most advantaged)	19 (10.6)	80 (29.2)	99 (21.9)	< 0.001
Census tract ICE-Race/Income, *n* (%)**				< 0.001
Q1^††^ (most disadvantaged)	65 (36.3)	17 (6.2)	82 (18.1)	< 0.001
Q2	54 (30.2)	58 (21.2)	112 (24.7)	0.300
Q3^†^	19 (10.6)	58 (21.2)	77 (17.0)	0.035
Q4	33 (18.4)	61 (22.3)	94 (20.8)	1.000
Q5^††^ (most advantaged)	8 (4.5)	80 (29.2)	88 (19.4)	< 0.001

^a^Early stage includes stages 1-3a and late stage includes stages 3b-4

*Significant at *p* < 0.05

**Significant at *p* < 0.001

^†^Post hoc test significant at *p* < 0.05

^††^Significant at *p* < 0.001

Multivariate and univariate regression analyses for both fixed and mixed effects models (with a random tract-level intercept) were tested. For both ICE-Income and ICE-Race/Income, the fixed effects only and mixed effects models produced similar coefficient results with no change in inference. However, the ICE-Race mixed effects model resulted in a singular fit and coefficients could not be estimated. This was likely due to insufficient between tract variation to support estimation of a tract-level random effect. With no covariates in the model, the variance of the random effect (measuring between-tract variance) was significant but small (var = 0.092, *p* = 0.014), which was reduced and became non-significant in most models once covariates were included (see Additional file 1: Table S1). Correspondingly, the intraclass correlation coefficient (ICC) for the census tract random effect was 0.027, indicating that only 2.7% of the total variance was attributed to between-tract variability. Given the similarity of results across fixed effects only and mixed effects models for ICE-Income and ICE-Race/Income and the singular fit for the ICE-Race random effects model, and the similarity of results between the fixed effects univariate and multivariate models for ICE-Race, results from the multilevel fixed effects only models are presented here for ease of interpretation (see Additional file 1: Table S2 for the full set of available random effects model results).

Table 3 shows the results of multilevel fixed-effects only models that separately test each ICE measure. In univariate models, increasing age of diagnosis was associated with lower odds of TNBC (OR: 0.93, 95% CI 0.89, 0.96), while Black race was associated with more than double the odds of TNBC relative to White race (OR: 2.48, 95% CI 2.01, 3.05). Models run with the insurance variable excluded patients age 65 and older and those with Medicare or unknown insurance to better model insurance as a proxy for health care access and SES (i.e., patients are eligible for Medicare beginning at age 65 regardless of SES). Insurance type was not significantly associated with TNBC, and therefore, the multivariate models were run for the full study population (i.e., including patients age 65 and older) and without the insurance status covariate. Across multivariate models for each ICE measure, increasing age of diagnosis and Black race were significantly associated with lower and greater odds of TNBC, respectively (*p*-values < 0.05). Quintiles Q1–Q4 of ICE-Race (corresponding to greater disadvantage relative to Q5) were associated with significantly higher odds of TNBC, even after adjustment for patient-level race and age of diagnosis, ranging from Q1 (AOR: 2.09) to Q4 (AOR: 1.76). Neither ICE-Income nor ICE-Race/Income quintiles were significantly associated with TNBC after covariate adjustment, though their adjusted odds ratios suggested positive associations with TNBC. No significant interactions were observed in models that included cross-level interaction terms between patient-level race and tract-level ICE (Additional file 1: Table S3).

**Table 3:** Odds of triple negative breast cancer by age, race, insurance, and census tract ICE-Race, -Income, and -Race/Income

	Breast cancer patients (*N* = 3316)			
	Univariate OR, 95% CI	Multivariate (ICE-Race) AOR, 95% CI	Multivariate (ICE-Income) AOR, 95% CI	Multivariate (ICE-Race/Income) AOR, 95% CI
Age at diagnosis^a^	0.93 (0.89, 0.96)**	0.94 (0.91, 0.98)*	0.94 (0.90, 0.98)*	0.94 (0.91, 0.98)*
Race (ref = White)				
Black/African American	2.48 (2.01, 3.05)**	2.02 (1.59, 2.58)**	2.21 (1.77, 2.77)**	2.18 (1.72, 2.77)**
Insurance (ref = commercial)				
Medicaid/None^b^	1.29 (0.83, 1.95)	–	–	–
Census tract ICE-Race (ref = Q5)				
Q1 (most disadvantaged)	3.45 (2.41, 4.99)**	2.09 (1.40, 3.13)**	–	–
Q2	2.12 (1.49, 3.06)**	1.65 (1.15, 2.41)*	–	–
Q3	1.81 (1.27, 2.60)*	1.54 (1.08, 2.23)*	–	–
Q4	1.86 (1.30, 2.69)**	1.76 (1.23, 2.54)*	–	–
Census tract ICE-Income (ref = Q5)				
Q1 (most disadvantaged)	1.90 (1.38, 2.61)**	–	1.33 (0.95, 1.87)	–
Q2	1.68 (1.24, 2.27)**	–	1.36 (1.00, 1.86)	–
Q3	1.15 (0.82, 1.60)	–	1.10 (0.78, 1.54)	–
Q4	1.39 (1.04, 1.85)*	–	1.30 (0.97, 1.74)	–
Census tract ICE-Race/Income (ref = Q5)				
Q1 (most disadvantaged)	2.07 (1.49, 2.88)**	–	–	1.23 (0.85, 1.78)
Q2	1.70 (1.26, 2.30)**	–	–	1.31 (0.96, 1.80)
Q3	1.14 (0.82, 1.58)	–	–	1.07 (0.77, 1.48)
Q4	1.13 (0.83, 1.54)	–	–	1.01 (0.74, 1.38)

^a^OR and AORs correspond to 5-year increases in age

^b^OR for insurance is from model that excludes patients age 65 and up and those with Medicare insurance in order to better model insurance as a proxy measure for socioeconomic status (*N* = 1691)

*Significant at *p* < 0.05

**Significant at *p* < 0.001

The multilevel fixed-effects only model of age and ICE-Race was stratified by patient-level race to further characterize the relationship between patient race, area-level segregation, and TNBC (Table 4). Increasing age of diagnosis was associated with decreased odds of TNBC for Black patients (AOR: 0.89, 95% CI 0.83, 0.95) but not White patients (AOR: 0.97, 95% CI 0.92, 1.01). Among Black patients, ICE-Race quintiles were no longer associated with TNBC (*p*-values > 0.05). The magnitude of the adjusted odds ratios suggested a positive association, which the relatively small Black patient sample (*N* = 776) may be underpowered to detect. ICE-Income and -Race/Income quintiles were also not associated with TNBC for Black patients (*p*-values > 0.05). Among White patients, ICE-Race quintiles Q1, Q2, and Q4 were associated with significantly greater odds of TNBC (*p*-values < 0.05). This result would suggest that White women living in predominantly Black census tracts were more likely to be diagnosed with TNBC, relative to other forms of invasive breast cancer, compared to White women living in predominantly White census tracts. For ICE-Income, only quintile Q2 was significantly associated with TNBC (AOR: 1.52, 95% CI 1.05, 2.20), with a non-significant trend in the expected direction observed for Q1 (AOR: 1.25, 95% CI 0.76, 2.01). This would suggest that White women living in low-income census tracts are at an elevated risk for TNBC, relative to other forms of invasive breast cancer among White women living in higher-income census tracts. No significant associations with TNBC were observed for ICE-Race/Income among White women.

**Table 4:** Odds of triple negative breast cancer by age and census tract ICE-Race, -Income, and -Race/Income, stratified by race

	Breast cancer patients (Black *N* = 776, White *N* = 2540)	
	Black patients, multivariate AOR, 95% CI	White patients, multivariate AOR, 95% CI
*Model 1: Age and ICE-Race*		
Age at diagnosis^a^	0.89 (0.83, 0.95)**	0.97 (0.92, 1.01)
Census tract ICE-Race (ref = Q5)		
Q1 (most disadvantaged)	2.90 (0.98, 12.42)	2.53 (1.49, 4.22)**
Q2	2.40 (0.80, 10.40)	1.62 (1.07, 2.45)*
Q3	2.78 (0.91, 12.12)	1.40 (0.94, 2.08)
Q4	2.63 (0.78, 12.15)	1.71 (1.17, 2.52)*
*Model 2: Age and ICE-Income*		
Age at diagnosis^a^	0.89 (0.83, 0.95)**	0.96 (0.92, 1.01)
Census tract ICE–Income (ref = Q5)		
Q1 (most disadvantaged)	1.50 (0.85, 2.76)	1.25 (0.76, 2.01)
Q2	1.22 (0.67, 2.28)	1.52 (1.05, 2.20)*
Q3	1.76 (0.88, 3.56)	0.94 (0.63, 1.39)
Q4	1.44 (0.78, 2.72)	1.27 (0.91, 1.77)
*Model 3: Age and ICE-Race/Income*		
Age at diagnosis^a^	0.89 (0.83, 0.95)**	0.97 (0.92, 1.01)
Census tract ICE-Race/Rncome (ref = Q5)		
Q1 (most disadvantaged)	1.81 (0.84, 4.34)	1.25 (0.70, 2.15)
Q2	1.84 (0.85, 4.47)	1.30 (0.91, 1.87)
Q3	1.68 (0.69, 4.40)	1.02 (0.71, 1.45)
Q4	1.83 (0.81, 4.57)	0.89 (0.63, 1.27)

^a^AORs correspond to 5-year increases in age

*Significant at *p* < 0.05

**Significant at *p* < 0.001

**Fig. 1:**
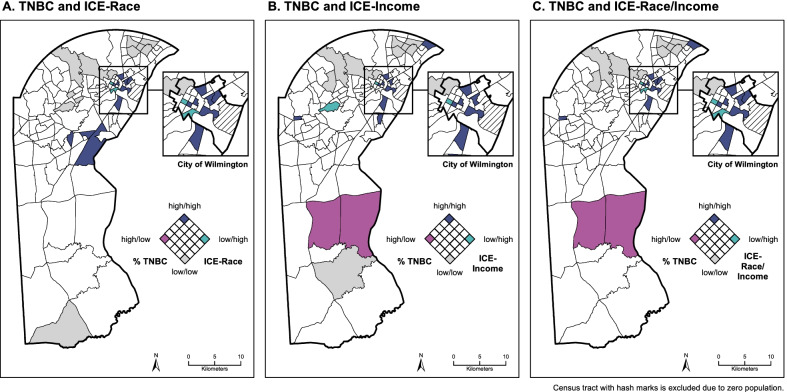
Spatial covariation of triple negative breast cancer (TNBC) prevalence and ICE measures, New Castle County, DE. **A**–**C** depict quintiles of TNBC prevalence and ICE-measured disadvantage (by race, income, and race/income) at the census tract level in New Castle County, DE. The extremes of the classification system in light gray, magenta, teal, and blue represent the spatial covariation of both measures, ranging from lower in both to higher in both. Across all maps, higher TNBC prevalence and higher ICE-measured disadvantage overlap in the City of Wilmington, as shown by the blue tracts. ICE-Race (**A**) appears to correlate with TNBC more strongly than ICE-Income (**B**) or ICE-Race/Income (**C**), as map **A** has more tracts classified as low–low (light gray) or high–high (blue) in both measures. ICE-Race (**A**) correlates with higher TNBC prevalence in additional census tracts south of the City of Wilmington, which correspond to tracts that have relatively large Black populations but relatively less income deprivation measured by ICE (**B**, **C**)

Figure 1 shows the spatial covariation of TNBC prevalence and ICE measures across county census tracts. Tracts are symbolized as follows: (1) light gray—lower% TNBC and lower ICE-measured disadvantage; (2) magenta—higher% TNBC and lower ICE-measured disadvantage; (3) teal—lower% TNBC and higher ICE-measured disadvantage, and (4) blue—higher% TNBC and higher ICE-measured disadvantage. TNBC prevalence appears to correlate most strongly with ICE-Race, represented by a greater number of dark blue census tracts in Fig. 1A relative to ICE-Income and ICE-Race/Income shown in Fig. 1B, C, respectively. Across all maps, higher TNBC prevalence and higher ICE-measured disadvantage overlap in the greater Wilmington area, extending southwest and in the northeastern-most corner of the county. Figure 1B (ICE-Income) and Fig. 1C (ICE-Race/Income) each depict two census tracts with higher TNBC prevalence but lower ICE-measured disadvantage in the southern part of the county. Each of the maps depict two census tracts with lower TNBC prevalence but higher ICE-measured disadvantage in the north-central part of the county.

The tract classifications based on quintiles of TNBC prevalence and ICE-Race appear to differentiate the affected populations and area-level systems of exposure (Table 5). As expected, the ten census tracts characterized as high in both TNBC and ICE-Race disadvantage (“high/high”) had a 28.8% prevalence of TNBC among breast cancer patients, and 63.9% of their general population from these census tracts were Black. Compared to the 12 tracts that were characterized as both low in TNBC and ICE-Race disadvantage (“low/low”), residents living in the high/high tracts had greater rates of poverty (23.3% vs. 5.0%) and higher rates of completing less than a high school education (13.0% vs. 5.6%). Comparing high/high and low/low tracts, the former had more than double the count (19 vs. 8) and density (0.48 vs. 0.20) of alcohol retailers. Similar but weaker patterns were observed for fast-food retailer counts (14 vs. 12) and density (0.35 vs. 0.30). High/high tracts also had greater prevalence of AUD (25.4% vs. 14.9%) and obesity (43.4% vs. 33.9%). Additional file 1: Tables S4 and S5 present these descriptive statistics according to classifications based on ICE-Income and ICE-Race/Income, respectively, which were similar to those based on ICE-Race. Additional file 2: Figure S1 shows the distribution of alcohol and fast-food retailers and AUD and obesity prevalence by tract ICE quintiles.

**Table 5:** Census tract characteristics by TNBC prevalence and race-ICE quintiles

	Lower TNBC, lower race-ICE disadvantage (population *N* = 39,924, tract *N* = 12)^a^	Lower TNBC, higher race-ICE disadvantage (population *N* = 4989, tract *N* = 2)^b^	Higher TNBC, lower race-ICE disadvantage (tract *N* = 0)^c^	Higher TNBC, higher race-ICE disadvantage (population *N* = 39,647, tract *N* = 10)^d^
% TNBC^e^	4.1%	0.0%	–	28.8%
ICE-Race, mean (SD)^f^	0.82 (0.07)	− 0.37 (0.16)	–	− 0.48 (0.33)
% Black^f^	4.5%	56.0%	–	63.9%
% Poverty^f^	5.0%	27.0%	–	23.3%
% Without high school education^f,g^	5.6%	15.4%	–	13.0%
Alcohol retailers	8	4	–	19
Fast-food retailers	12	5	–	14
Alcohol retailers per 1000 people	0.20	0.80	–	0.48
Fast-food retailers per 1000 people	0.30	1.00	–	0.35
% With AUD^h^	14.9%	23.9%	–	25.4%
% With obesity^h^	33.9%	38.7%	–	43.4%

^a^Corresponds to light gray tracts in Fig. 1A

^b^Corresponds to teal tracts in Fig. 1A

^c^Corresponds to magenta tracts in Fig. 1A

^d^Corresponds to blue tracts in Fig. 1A

^e^TNBC prevalence determined from patients diagnosed with invasive breast cancer at HFGCCRI between 2012 and 2020 (*N* = 3449)

^f^Census tract population data from American Community Survey 5-year estimates, 2014–2018

^g^Educational attainment defined for the population aged 25 and older

^h^AUD and obesity prevalence determined from adults hospitalized at Christiana Care between July 1, 2018 and June 30, 2019 (*N* = 20,310)

## Discussion

In a cohort of breast cancer patients from New Castle County, Delaware, a geographic area with among the highest rates of TNBC in the US, we tested whether different versions of the ICE metric could efficiently identify census tracts with greater odds of TNBC relative to other invasive subtypes of breast cancer. Consistent with prior epidemiological findings [13], women with TNBC were younger, twice as likely to be Black, more likely to have Medicaid or no insurance, and twice as likely to present with a late-stage cancer. As hypothesized, ICE-Race, -Income, and ICE-Race/Income metrics were associated with the odds of TNBC on a bivariate basis. However, contrary to our hypotheses, only the ICE-Race metric was significantly associated with higher odds of TNBC in multilevel models that adjusted for patient-level age and race. To our knowledge, this is the first multilevel study that evaluated the use of ICE metrics in the context of TNBC.

By including both patient- and census tract-level measures of race, our results help to clarify the extent to which the higher rates of TNBC observed in predominantly Black neighborhoods can be attributed to compositional or contextual effects. That is, if the relationship between ICE-Race and census tract odds of TNBC became nonsignificant when adjusting for patient-level race, we might infer that the apparent neighborhood effects were likely an artifact of the neighborhood composition. By contrast, our results suggest that the relationship between TNBC and area-level measures of race are a function of both composition and context. Stated differently, compared to predominantly White neighborhoods, predominantly Black neighborhoods may differ in some important ways that have relevance for TNBC risk. This interpretation is bolstered by the multilevel results stratified by patient-level race, which showed that White patients who lived in predominantly Black census tracts were also at greater odds of TNBC than White patients living in predominantly White census tracts.

These results are in partial disagreement with the findings from two prior studies, which found higher odds of TNBC for Black women living in lower-SES, predominantly White neighborhoods [15, 64]. The authors of these reports reasoned that Black women who reside in predominantly Black neighborhoods may have social support systems that mitigate the effects of living in a low-SES environment. However, the potentially protective effects of social support derived from racial/ethnic enclaves on cancer outcomes has not been well-studied in Black populations compared to Hispanic or Asian populations. Not only did we find that census tracts with a higher proportion of Black residents were associated with higher odds of TNBC, we also did not observe any significant patient-level race by ICE-Income cross-level interactions. Our results are more consistent with a segregation-based disparate exposure hypothesis. That is, through a series of historical laws and policies (e.g., “redlining”), the US, state, and local governments forcibly segregated communities by race and denied these marginalized communities access to financing and other forms of investment, a residential pattern that largely persists through present day [65–67]. The effect of these segregationist policies can be measured in terms of disparate exposures (e.g., increased concentration of alcohol retailers) [68, 69], poorer access to healthy food [70], and ultimately worse cancer and other health outcomes [71–73]. Indeed, we observed higher counts of alcohol and fast-food retailers, and correspondingly higher rates of AUD and obesity, in census tracts that were categorized into the most disadvantaged ICE-Race quintile and had the highest odds of TNBC.

This study was limited by its single-site, cross-sectional design. Findings may not be generalizable to populations from other geographic areas. Nevertheless, given the notably elevated rates of TNBC in Delaware, the results of this study can help to inform local cancer control and prevention efforts while providing a methodological proof of concept that can be replicated for other geographic areas. Without patient residential histories, it is unclear to what degree prior neighborhood exposures may have been associated with TNBC risk. Furthermore, while we did have access to patient-level measures of race and insurance status, we did not have patient-level measures of other relevant exposures (e.g., alcohol use) and were not able to determine ethnicity (Hispanic vs. non-Hispanic) at the patient level. Further, we limited our investigation of neighborhood to include ICE measures, given there is no standard set of measures used to measure neighborhood deprivation. It is possible other socioeconomic indices (e.g., Yost index, ADI) could also provide additional insights into the impact of neighborhood on TNBC [74]. Future research should be conducted on cohorts from a range of geographic areas, with more detailed patient- and area-level measures of exposure, to further characterize the multilevel relationships between race, SES, and TNBC.

## Conclusions

This study provides preliminary evidence to suggest that the ICE-Race metric can efficiently identify census tracts with higher odds of TNBC due to both compositional and contextual effects. Preliminary evidence also suggests that the contextual effects may be driven, at least in part, by potentially modifiable metabolic exposures, such as alcohol use and obesity. Krieger and colleagues have called for including ICE metrics in cancer registries to facilitate the monitoring of cancer inequities [29]. Going further, the use of ICE metrics can help to advance the study of racial disparities in breast cancer from a methodology based on traditional risk factors to one grounded in a causal architecture framework. Rather than studying individual risk factors in isolation without considering neighborhood effects, the use of large and representative pooled patient cohorts can be employed to evaluate the multilevel, multifactorial relationships between exposures and TNBC. Such efforts could be complemented by basic and translational research designed to delineate mechanisms of pathophysiology and facilitate biomarker discovery. Together, these lines of research could inform risk stratification approaches to improve early detection, more effectively target risk factor modification interventions to the communities at greatest risk, and advance health equity [75, 76].

## Supplementary Information

**Additional file 1: Tables S1**. Variance of census tract random effect for breast cancer subtype (triple negative breast cancer vs. not), before and after covariate adjustment. **Table S2**. Comparison of fixed- and mixed-effects models for odds of triple negative breast cancer by age and census tract race-, income-, and race/income ICE. **Table S3**. Odds of triple negative breast cancer by race and census tract ICE interactions. **Table S4**. Census tract characteristics by TNBC prevalence and income-ICE quintiles. **Table S5**. Census tract characteristics by TNBC prevalence and ICE-Race/Income quintiles.

**Additional file 2: Figure S1**. Retail exposures and comorbidity prevalence by ICE quintiles, New Castle County, DE. Shows place-based systems of exposure related to metabolic risk factors for triple negative breast cancer (TNBC) by census tract ICE quintiles in New Castle County, DE. Alcohol retailers (A) and alcohol use disorder prevalence (C) show a graded relationship with ICE, with both measures highest in tracts classified as Q1 (greatest ICE-measured disadvantage). Fast-food retailers (B) are most prevalent in Q3–Q4 ICE tracts, while obesity prevalence (D) varies little by ICE. All measures show similar variation by race-, income-, and race/income-ICE.

## Data Availability

Datasets generated for this study from the HFGCCRI cancer registry or the Christiana Care Health System EHR are not publicly available because they contain protected health information but may be made available in a deidentified format from the corresponding author on reasonable request. Census tract datasets generated for this study based on publicly available sources, including ICE metrics and the alcohol and fast-food retailer locations, are available from the corresponding author on reasonable request.
