# Supplementary material for: Racial disparities in triple negative breast cancer: toward a causal architecture approach
Source: Breast Cancer Res. 2022 Jun 1;24:37. doi: 10.1186/s13058-022-01533-z (PMC9158353; doi:10.1186/s13058-022-01533-z)
Supplement: Supplementary file 1 — Additional file 1: Tables S1. Variance of census tract random effect for breast cancer subtype (triple negative breast cancer vs. not), before and after covariate adjustment. Table S2. Comparison of fixed- and mixed-effects models for odds of triple negative breast cancer by age and census tract race-, income-, and race/income ICE. Table S3. Odds of triple negative breast cancer by race and census tract ICE interactions. Table S4. Census tract characteristics by TNBC prevalence and income-ICE quintiles. Table S5. Census tract characteristics by TNBC prevalence and ICE-Race/Income quintiles. [file 13058_2022_1533_MOESM1_ESM.docx]

**Additional file 1: Tables**

See Additional file 1: Table S1 for variance estimates for the ICE-Race univariate and multivariate models.

See Additional file 1: Table S2 for the full sets of fixed and mixed effects models.

See Additional file 1: Table S3 for models that included cross-level interaction terms between patient-level race and tract-level ICE.

See Additional file 1: Table S4. for census tract characteristics by TNBC prevalence and ICE-Income quintiles and Supplemental Table 4 for census tract characteristics by TNBC prevalence and ICE-Race/Income quintiles.

See Additional file 1: Table S5 for census tract characteristics by TNBC prevalence and ICE-Race/Income quintiles

Additional file 1: Table S1. Variance of census tract random effect for breast cancer subtype (triple negative breast cancer vs. not), before and after covariate adjustment

|  | Breast cancer patients  (*N*=3316) |
| --- | --- |
|  | Univariate models:  census tract random effect,  variance (p-value) |
| No covariates | 0.092 (0.014)* |
| Age at diagnosis | 0.079 (0.017)* |
| Race | 0.002 (0.417) |
| Insurance^a^ | 0.055 (0.173) |
| Census tract ICE-Race quintiles | x |
| Census tract ICE-Income quintiles | 0.054 (0.041)* |
| Census tract ICE-Race/Income quintiles | 0.032 (0.112) |

^a^Census tract random effect variance adjusted for insurance (commercial, Medicaid/none) is from model that excludes patients age 65 and up and those with Medicare insurance in order to better model insurance as a proxy measure for socioeconomic status (*N*=1691).

x. Census tract random effect variance not reported due to singular model fit.

*Significant at p<0.05, **significant at p<0.001

Additional file 1: Table S2. Comparison of fixed- and mixed-effects models for odds of triple negative breast cancer by age and census tract race-, income-, and race/income ICE

|  | Breast cancer patients  (*N*=3316) | | | |
| --- | --- | --- | --- | --- |
|  | Fixed effects univariate  OR, 95% CI | Mixed effects univariate  OR, 95% CI | Fixed effects multivariate  AOR, 95% CI | Mixed effects multivariate  AOR, 95% CI |
| **Model 1: age and ICE-Race** | | | | |
| Age at diagnosis^a^ | 0.93 (0.89, 0.96)** | 0.93 (0.89, 0.96)** | 0.93 (0.90, 0.97)** | x |
| Census tract ICE-Race (ref=Q5) | | | | |
| Q1 (most disadvantaged) | 3.45 (2.41, 4.99)** | x | 3.30 (2.31, 4.79)** | x |
| Q2 | 2.12 (1.49, 3.06)** | x | 2.03 (1.42, 2.93)** | x |
| Q3 | 1.81 (1.27, 2.60)* | x | 1.74 (1.22, 2.50)* | x |
| Q4 | 1.86 (1.30, 2.69)** | x | 1.81 (1.27, 2.62)* | x |
| **Model 2: age and ICE-Income** | | | | |
| Age at diagnosis^a^ | 0.93 (0.89, 0.96)** | 0.93 (0.89, 0.96)** | 0.93 (0.89, 0.96)** | 0.93 (0.89, 0.96)** |
| Census tract ICE-Income (ref=Q5) | | | | |
| Q1 (most disadvantaged) | 1.90 (1.38, 2.61)** | 1.97 (1.38, 2.80)** | 1.90 (1.38, 2.62)** | 1.96 (1.38, 2.77)** |
| Q2 | 1.68 (1.24, 2.27)** | 1.70 (1.22, 2.38)* | 1.68 (1.24, 2.27)** | 1.70 (1.22, 2.35)* |
| Q3 | 1.15 (0.82, 1.60) | 1.16 (0.81, 1.67) | 1.15 (0.82, 1.61) | 1.16 (0.82, 1.66) |
| Q4 | 1.39 (1.04, 1.85)* | 1.41 (1.02, 1.95)* | 1.42 (1.06, 1.89)* | 1.43 (1.04, 1.96)* |
| **Model 3: age and ICE-Race/Income** | | | | |
| Age at diagnosis^a^ | 0.93 (0.89, 0.96)** | 0.93 (0.89, 0.96)** | 0.93 (0.90, 0.96)** | 0.93 (0.90, 0.97)** |
| Census tract ICE-Race/Income (ref=Q5) | | | | |
| Q1 (most disadvantaged) | 2.07 (1.49, 2.88)** | 2.10 (1.48, 2.97)** | 2.03 (1.46, 2.82)** | 2.05 (1.45, 2.89)** |
| Q2 | 1.70 (1.26, 2.30)** | 1.71 (1.24, 2.35)* | 1.67 (1.24, 2.26)** | 1.67 (1.22, 2.30)* |
| Q3 | 1.14 (0.82, 1.58) | 1.14 (0.81, 1.61) | 1.15 (0.83, 1.59) | 1.15 (0.82, 1.62) |
| Q4 | 1.13 (0.83, 1.54) | 1.13 (0.81, 1.57) | 1.12 (0.82, 1.53) | 1.12 (0.81, 1.56) |

^a^AORs correspond to 5-year increases in age.

*Significant at p<0.05, **significant at p<0.001

^x^Singular model fit; results not reported.

Additional file 1: Table S3. Odds of triple negative breast cancer by race and census tract ICE interactions

|  | Breast cancer patients  (*N*=3316) | | | |
| --- | --- | --- | --- | --- |
|  | Univariate  OR, 95% CI | Multivariate  (race ICE)  AOR, 95% CI | Multivariate  (income ICE)  AOR, 95% CI | Multivariate (race/income ICE)  AOR, 95% CI |
| Age at diagnosis^a^ | 0.93 (0.89, 0.96)** | 0.94 (0.91, 0.98)* | 0.94 (0.90, 0.97)** | 0.94 (0.91, 0.98)* |
| Race (ref=White) | | | | |
| Black/African American | 2.48 (2.01, 3.05)** | 1.31 (0.31, 3.91) | 2.05 (1.16, 3.50)* | 1.46 (0.62, 3.06) |
| Insurance (ref=commercial) | | | | |
| Medicaid/none^b^ | 1.29 (0.83, 1.95) | -- | -- | -- |
| Census tract ICE-Race (ref=Q5) | | | | |
| Q1 (most disadvantaged) | 3.45 (2.41, 4.99)** | 2.51 (1.48, 4.19)** | -- | -- |
| Q2 | 2.12 (1.49, 3.06)** | 1.60 (1.06, 2.42)* | -- | -- |
| Q3 | 1.81 (1.27, 2.60)* | 1.38 (0.93, 2.05) | -- | -- |
| Q4 | 1.86 (1.30, 2.69)** | 1.69 (1.16, 2.50)* | -- | -- |
| Race * ICE-Race (refs=Black, Q5) | | | | |
| Black * Q1 | -- | 1.16 (0.35, 5.34) | -- | -- |
| Black * Q2 | -- | 1.55 (0.47, 7.01) | -- | -- |
| Black * Q3 | -- | 2.02 (0.62, 9.20) | -- | -- |
| Black * Q4 | -- | 1.61 (0.45, 7.69) | -- | -- |
| Census tract ICE-income (ref=Q5) | | | | |
| Q1 (most disadvantaged) | 1.90 (1.38, 2.61)** | -- | 1.26 (0.76, 2.02) | -- |
| Q2 | 1.68 (1.24, 2.27)** | -- | 1.54 (1.06, 2.22)* | -- |
| Q3 | 1.15 (0.82, 1.60) | -- | 0.95 (0.63, 1.40) | -- |
| Q4 | 1.39 (1.04, 1.85)* | -- | 1.28 (0.92, 1.78) | -- |
| Race * ICE-Income  (refs=Black, Q5) | | | | |
| Black * Q1 | -- | -- | 1.15 (0.55, 2.50) | -- |
| Black * Q2 | -- | -- | 0.80 (0.40, 1.65) | -- |
| Black * Q3 | -- | -- | 1.79 (0.81, 4.00) | -- |
| Black * Q4 | -- | -- | 1.09 (0.54, 2.22) | -- |
| Census tract ICE-Race/Income  (ref=Q5) | | | | |
| Q1 (most disadvantaged) | 2.07 (1.49, 2.88)** | -- | -- | 1.26 (0.70, 2.15) |
| Q2 | 1.70 (1.26, 2.30)** | -- | -- | 1.30 (0.90, 1.86) |
| Q3 | 1.14 (0.82, 1.58) | -- | -- | 1.02 (0.71, 1.46) |
| Q4 | 1.13 (0.83, 1.54) | -- | -- | 0.89 (0.63, 1.27) |
| Race * ICE-Race/Income  (refs=Black, Q5) | | | | |
| Black * Q1 | -- | -- | -- | 1.39 (0.54, 3.90) |
| Black * Q2 | -- | -- | -- | 1.40 (0.60, 3.600 |
| Black * Q3 | -- | -- | -- | 1.55 (0.60, 4.31) |
| Black * Q4 | -- | -- | -- | 1.98 (0.82, 5.24) |

^a^OR and AORs correspond to 5-year increases in age.

^b^OR for insurance is from model that excludes patients age 65 and up and those with Medicare insurance in order to better model insurance as a proxy measure for socioeconomic status (*N*=1691).

*Significant at p<0.05, **significant at p<0.001

Additional file 1: Table S4. Census tract characteristics by TNBC prevalence and income-ICE quintiles

|  | Lower TNBC,  lower ICE-Income disadvantage  (population *N*=43412, tract *N*=10)^a^ | Lower TNBC,  higher ICE-Income disadvantage  (population *N*=6231, tract *N*=2)^b^ | Higher TNBC,  lower ICE-Income disadvantage  (population *N*=14524, tract *N*=2)^c^ | Higher TNBC,  higher ICE-Income disadvantage  (population *N*=27624, tract *N*=9)^d^ |
| --- | --- | --- | --- | --- |
| % TNBC^e^ | 4.3% | 2.4% | 20.0% | 26.4% |
| Income-ICE, mean (SD)^f^ | 0.36 (0.06) | -0.15 (0.05) | 0.40 (0.01) | -0.29 (0.17) |
| % Black^f^ | 8.6% | 24.2% | 14.7% | 56.2% |
| % poverty^f^ | 3.6% | 18.9% | 3.4% | 32.3% |
| % without high school education^f,g^ | 4.4% | 15.7% | 4.8% | 14.2% |
| Alcohol retailers | 5 | 5 | 2 | 16 |
| Fast-food retailers | 6 | 4 | 1 | 9 |
| Alcohol retailers per 1000 people | 0.12 | 0.80 | 0.14 | 0.58 |
| Fast-food retailers per 1000 people | 0.14 | 0.64 | 0.07 | 0.33 |
| % with AUD^h^ | 13.1% | 21.4% | 10.2% | 28.4% |
| % with obesity^h^ | 31.9% | 43.5% | 39.3% | 45.3% |

^a^Corresponds to light grey tracts in figure 1B

^b^Corresponds to teal tracts in figure 1B

^c^Corresponds to magenta tracts in figure 1B

^d^Corresponds to dark purple tracts in figure 1B

^e^TNBC prevalence determined from patients diagnosed with invasive breast cancer at HFGCCRI between 2012-2020 (*N*=3449)

^f^Census tract population data from American Community Survey 5-year estimates, 2014-2018

^g^Educational attainment defined for the population aged 25 and older

^h^AUD and obesity prevalence determined from adults hospitalized at Christiana Care between July 1, 2018 and June 30, 2019 (*N*=20310)

Additional file 1: Table S5. Census tract characteristics by TNBC prevalence and ICE-Race/Income quintiles

|  | Lower TNBC,  lower ICE-Race/Income disadvantage  (population *N*=35754, tract *N*=9)^a^ | Lower TNBC,  higher ICE-Race/Income disadvantage  (population *N*=4989, tract *N*=2)^b^ | Higher TNBC,  lower ICE-Race/Income disadvantage  (population *N*=14524, tract *N*=2)^c^ | Higher TNBC,  higher ICE-Race/Income disadvantage  (population *N*=26950, tract *N*=9)^d^ |
| --- | --- | --- | --- | --- |
| % TNBC^e^ | 3.9% | 0.0% | 20.0% | 29.0% |
| Race/income-ICE, mean (SD)^f^ | 0.37 (0.07) | -0.12 (0.12) | 0.34 (0.02) | -0.21 (0.17) |
| % Black^f^ | 5.6% | 56.0% | 14.7% | 64.5% |
| % poverty^f^ | 3.4% | 27.0% | 3.4% | 33.5% |
| % without high school education^f,g^ | 4.0% | 15.4% | 4.8% | 14.2% |
| Alcohol retailers | 5 | 4 | 2 | 17 |
| Fast-food retailers | 5 | 5 | 1 | 10 |
| Alcohol retailers per 1000 people | 0.14 | 0.80 | 0.14 | 0.63 |
| Fast-food retailers per 1000 people | 0.14 | 1.00 | 0.07 | 0.37 |
| % with AUD^h^ | 13.7% | 23.9% | 10.2% | 29.1% |
| % with obesity^h^ | 31.0% | 38.7% | 39.3% | 44.5% |

^a^Corresponds to light grey tracts in figure 1C

^b^Corresponds to teal tracts in figure 1C

^c^Corresponds to magenta tracts in figure 1C

^d^Corresponds to dark purple tracts in figure 1C

^e^TNBC prevalence determined from patients diagnosed with invasive breast cancer at HFGCCRI between 2012-2020 (*N*=3449)

^f^Census tract population data from American Community Survey 5-year estimates, 2014-2018

^g^Educational attainment defined for the population aged 25 and older

^h^AUD and obesity prevalence determined from adults hospitalized at Christiana Care between July 1, 2018 and June 30, 2019 (*N*=20310)
