# Supplementary figures and images for: Racial disparities in triple negative breast cancer: toward a causal architecture approach
Source: Breast Cancer Res. 2022 Jun 1;24:37. doi: 10.1186/s13058-022-01533-z (PMC9158353; doi:10.1186/s13058-022-01533-z)

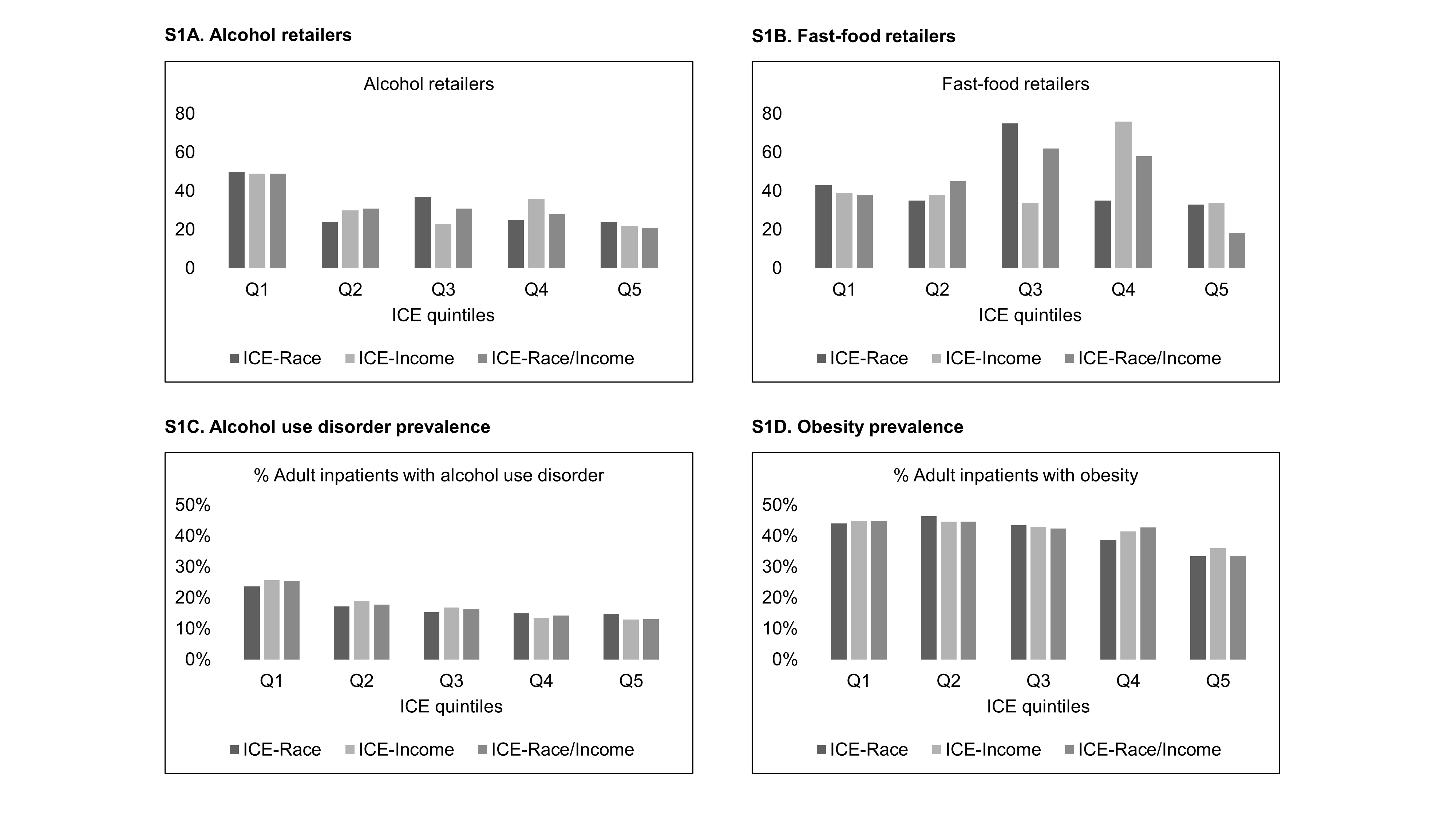

Supplement: Supplementary file 2 — Additional file 2: Figure S1. Retail exposures and comorbidity prevalence by ICE quintiles, New Castle County, DE. Shows place-based systems of exposure related to metabolic risk factors for triple negative breast cancer (TNBC) by census tract ICE quintiles in New Castle County, DE. Alcohol retailers (A) and alcohol use disorder prevalence (C) show a graded relationship with ICE, with both measures highest in tracts classified as Q1 (greatest ICE-measured disadvantage). Fast-food retailers (B) are most prevalent in Q3–Q4 ICE tracts, while obesity prevalence (D) varies little by ICE. All measures show similar variation by race-, income-, and race/income-ICE. [file 13058_2022_1533_MOESM2_ESM.tif]
